# Persistent bone and muscle deficits after anterior cruciate ligament reconstruction: implications for post-traumatic osteoarthritis risk

**DOI:** 10.1302/2046-3758.157.BJR-2025-0481.R1

**Published:** 2026-07-09

**Authors:** Karl Morgan, Lilly Dietel, Antara Jain, Zak Sheehy, Shoji Leach, Molly Robb, James Murray, Sunny Deo, Dario Cazzola, Jean-Philippe Walhin

**Affiliations:** 1 Department for Health, University of Bath, Bath, UK; 2 Centre for Health and Injury and Illness Prevention in Sport (CHI2PS), Department for Health, University of Bath, Bath, UK; 3 Centre for Nutrition and Exercise Metabolism (CNEM), Department for Health, University of Bath, Bath, UK; 4 Centre for Sport, Exercise and Osteoarthritis Versus Arthritis, University of Bath, Bath, UK; 5 North Bristol NHS Trust, Bristol, UK; 6 Great Western Hospital NHS Trust, Swindon, UK; 7 Centre for the Analysis of Motion, Entertainment Research and Applications (CAMERA), University of Bath, Bath, UK

**Keywords:** Anterior cruciate ligament reconstruction, Osteoarthritis, Bone density, Muscle, Cluster analysis, Tissue Properties, anterior cruciate ligament reconstruction, Mechanical properties, strengths, thigh, Trabecular bone, post-traumatic osteoarthritis, cortical bone, muscle atrophy, shank, post-traumatic osteoarthritis

## Abstract

**Aims:**

Anterior cruciate ligament reconstruction (ACLR) is associated with persistent strength deficits and thigh muscle atrophy, which may contribute to post-traumatic osteoarthritis (PTOA). Other potential musculoskeletal alterations, such as changes in bone morphology and mechanical properties, are less well understood. No study has comprehensively examined multi-tissue deficits post-ACLR, limiting insight into potential structural contributors to long-term joint vulnerability. Consequently, this cross-sectional study investigated differences in muscle size and quality, bone morphology, and mechanical properties between the ACLR and contralateral uninjured leg. The influence of leg dominance on musculoskeletal outcomes in a control population and the identification of distinct recovery profiles in the ACLR group were explored.

**Methods:**

A total of 32 individuals with a history of ACLR (1 to 6 years post-surgery) and 32 matched uninjured controls underwent peripheral quantitative computerised tomography at the shank (66% tibial length) and thigh (20% and 50% femoral length). Outcomes included muscle cross-sectional area, muscle density, subcutaneous fat, cortical and trabecular bone area and density, and bone resistance to bending, torsion, and compression. Clustering techniques were applied to explore potential high- and low-recovery profiles.

**Results:**

The ACLR leg demonstrated reduced muscle size and density, smaller cortical bone area, lower trabecular density, and impaired resistance to bending and compressive forces in comparison to the uninjured contralateral leg. Clustering revealed a low-recovery profile in 69% of ACLR participants, characterized by widespread musculoskeletal deficits.

**Conclusion:**

Persistent musculoskeletal alterations after ACLR may reflect compromised structural integrity associated with PTOA risk. Recovery profiling may help identify those at greatest risk, informing targeted interventions to improve long-term joint health.

Cite this article: *Bone Joint Res* 2026;15(7):822–835.

## Article focus

- To investigate persistent multi-tissue musculoskeletal deficits (muscle size, density, bone morphology, and bone mechanical properties such as resistance to compression and bending) following anterior cruciate ligament reconstruction (ACLR).
- To determine whether observed asymmetries are injury-related by comparing ACLR participants with age-, sex-, and body composition-matched controls.
- To explore potential recovery phenotypes post-ACLR using clustering techniques.

## Key messages

- ACLR legs showed persistent deficits in muscle size, muscle density, cortical bone area, trabecular density, and bone mechanical properties (reduced resistance to compression and bending) compared with the contralateral uninjured leg.
- These deficits were not present in matched controls, suggesting injury-related rather than physiological asymmetries.
- Multi-site imaging of both the femur and tibia enabled a comprehensive evaluation, and recovery profiling identified a ‘low-recovery’ phenotype in 69% of ACLR participants, potentially highlighting those at elevated post-traumatic osteoarthritis risk.

## Strengths and limitations

- First comprehensive multi-tissue and multi-site (femur and tibia) analysis after ACLR, including bone mechanical properties.
- Inclusion of a matched control cohort and application of unsupervised clustering to identify recovery phenotypes.
- Cross-sectional design limits causal inference; findings require confirmation in longitudinal studies.

## Introduction

Anterior cruciate ligament (ACL) injuries are associated with a risk of developing a condition called post-traumatic osteoarthritis (PTOA)^1^ and subsequent total knee arthroplasty,^2^ and there is an urgent need to understand the mechanisms that underpin this trajectory. One key factor implicated in PTOA development is persistent knee extensor weakness, which has been proposed to contribute to joint degeneration through a failure of musculoskeletal structures to adequately protect the joint during loading.^3^ Cross-sectional studies support a positive association between knee extensor strength and cartilage health in both uninjured individuals^4^ and knee injury populations.^5,6^ Muscle function plays a crucial role in attenuating joint loads during activities of daily living, locomotion, and sport. When muscular contributions are compromised, the joint may be exposed to abnormal mechanical stress, potentially accelerating cartilage degradation^7^ and loss of function.^8^ Consequently, knee PTOA disease incidence and progression may be regulated to some extent by knee extensor strength.

Persistent quadriceps weakness following ACL reconstruction (ACLR) is well documented. A systematic review and meta-analysis reported that strength deficits are most pronounced shortly after surgery, but remain evident compared to healthy controls even beyond 12 months post-reconstruction.^9^ This suggests that the ‘new normal’ is lower knee extensor strength in the reconstructed leg in the years following injury and surgery. The purported causes of knee extensor weakness are arthrogenic inhibition,^9^ muscle atrophy,^10^ and graft type;^11^ however, muscle tissue size deficits are the least explored domain. In addition, emerging evidence suggests that muscle quality, which is related to intramuscular fat content^12^ and is typically defined as strength per unit of mass,^13^ may be an important contributor to strength outcomes post-ACLR.^14^ Intramuscular fat infiltration in the reconstructed leg is pertinent, given that in idiopathic knee OA populations, higher intramuscular fat tissue content is associated with accelerated joint tissue degeneration.^15,16^

In parallel with potential soft-tissue maladaptations, ACL injury may also affect bone tissue. Studies have reported lower bone mineral density (BMD) in the reconstructed leg.^17,18^ Notably, these studies have typically employed 2D analysis techniques such as dual-energy X-ray absorptiometry (DEXA), which does not distinguish cortical and trabecular bone compartments. Another unresolved question is whether bone geometry is adversely affected following ACLR, as few studies have considered potential changes in cortical or trabecular bone area. If both the density and geometry of bone tissues are reduced following ACLR, the mechanical properties of the bone tissue, such as the resistance to shear or compressive forces, may be altered. Although the role of bone health in PTOA development is still unclear, if altered densities and geometries exist, there are potential implications of altered bone metabolism influencing OA pathogenesis.^19,20^

To date, no studies have simultaneously evaluated soft-tissue, bone, and the mechanical properties of bone across multiple scan sites in both the thigh and shank in individuals at risk of PTOA. Consequently, the primary aim of this study was to investigate whether asymmetries exist between the reconstructed and uninjured contralateral legs in soft-tissue (muscle cross-sectional area (MCSA), muscle density (MD), subcutaneous adipose tissue area (SATA)), bone tissue (cortical bone area (CoA), cortical bone density (CoD), trabecular bone area (TraA), trabecular bone density (TraD)), and the biomechanical properties of bone (strength strain index (SSI), polar second moment of area (iPo), and compressive strength index (BSId)). The secondary aim was to examine the potential influence of leg dominance on morphological and mechanical outcomes following ACLR. The exploratory aim was to determine whether multivariate clustering could identify biologically distinct subgroups that may reflect differing levels of tissue integrity and, theoretically, could serve as a foundation for future longitudinal research into PTOA risk stratification. The primary hypothesis was that the reconstructed leg would exhibit significantly lower MCSA, MD, SATA, CoA, CoD, TraA, TraD, and reduced biomechanical bone properties (SSI, iPo, BSId) compared to the uninjured contralateral leg.

## Methods

### Participants

This observational cross-sectional study was approved by an NHS Research Ethics Committee (IRAS: 304181) and prospectively registered on ClinicalTrials.gov (NCT05306054). ACLR participants were aged between 18 and 45 years, had completed rehabilitation, and were medically cleared for physical activity. Exclusion criteria included bilateral knee injuries; musculoskeletal, cardiovascular, respiratory, immune, metabolic, or neurological disorders; pregnancy; or any condition precluding safe physical activity. Control participants were aged between 18 and 45 years, with no history of serious joint injuries (e.g. ligaments, menisci, cartilage, intra-articular fractures). Exclusion criteria matched the ACLR group, aside from injury history. Controls were matched to ACLR participants by sex, age (± 5 years), and BMI, prioritizing a strict threshold of ± 2.5 kg/m^2^ and BMI category (e.g. underweight, normal weight, overweight, obese). DEXA scans were conducted post-matching to compare fat and muscle mass. All participants provided informed electronic consent. The study adhered to the Declaration of Helsinki^21^ and was reported in accordance with STROBE guidelines (Supplementary Material 1).^22^ Based on a meta-analysis reporting moderate-to-large thigh muscle size deficits (*d* = 0.65), a sample of 29 was required (*α* = 0.017, 1-β = 0.8).^23^ A total of 32 ACLR and 32 matched control participants were recruited. Following online enrolment, participants completed a baseline questionnaire (demographics, injury history, leg dominance, and physical activity history, including the highest level of sport engaged in prior to injury). Among ACLR participants, 19% reported recreational-level sport, 44% regional/school-level, 28% national-level, and 9% international-level competition. One participant identified as a semi-professional athlete. Once completed, participants attended a laboratory visit at the University of Bath after an overnight fast, adequately hydrated, and having abstained from caffeine, alcohol, and moderate-to-vigorous physical activity in the previous 24 hours. During this visit, participants completed the Knee Injury and Osteoarthritis Outcome Score.^24^ Data collection occurred from July 2022 to March 2024.

### Leg morphology imaging

Participants underwent six peripheral quantitative computerised tomography (pQCT) scans (XCT 3000, Stratec Medizintechnik, Germany) at the shank (66% of tibial length from the distal growth plate) and thigh (20% and 50% of femoral length from the distal growth plate). The device demonstrates strong test-retest reliability and criterion validity for assessing MCSA,^25-28^ MD,^26-30^ and bone mechanical properties.^31^ A hydroxyapatite phantom was scanned before each session for quality assurance. Scans were acquired using proprietary software, and a qualitative rating scale was used during acquisition to screen for excessive motion or out-of-bounds artefacts.^32^ Scanner speed was set at 25 mm/s. Pixel spacing was set at 0.8 mm.

Bone parameters included cortical density (CoD), reflecting mineral content and cortical integrity; cortical area (CoA), representing cortical geometry; trabecular density (TraD), assessing trabecular mineralization; and trabecular area (TraA), representing trabecular geometry. A threshold of 280 mg/cm³ was applied to define the periosteal contour. For trabecular measurements, an inner threshold of 400 mg/cm³ was used, with cortical bone removed via sequential contour detection. For cortical analysis, a 690 mg/cm³ threshold was applied. A 3 × 3 filter was used to reduce noise and enhance image quality.

Mechanical properties of bone were estimated using SSI, iPo, and BSId, which collectively provide critical insights into musculoskeletal structure and function.^31^ SSI integrates bone density, cross-sectional geometry, and material distribution to estimate resistance to bending and torsional forces (Supplementary Material 2). iPo is a calculation of resistance to torsional and bending forces based on mass distribution around the central axis (Supplementary Material 2). BSId is a product of density and cross-sectional area, indicating compressive strength (Supplementary Material 2).

Soft-tissue variables measured using pQCT encompassed MCSA, which quantifies the size of skeletal muscle; MD, reflecting muscle quality and intra-muscular adipose tissue deposition; and SATA, representing the fat tissue within the subcutaneous compartment. pQCT images were exported from Stratec proprietary software and analyzed in Fiji^33^ using the pQCT plugin.^34^ Tissue segmentation was based on density thresholds: air (-40 mg/cm³), fat (40 mg/cm³), muscle (50 mg/cm³), and marrow (80 mg/cm³), with an overall soft-tissue threshold of 200 mg/cm³. A threshold of 550 mm² was used for area measurements, and 690 mg/cm³ was applied to distinguish bone from other tissues. A 7 × 7 filter was used to enhance soft-tissue boundaries and reduce image noise.

### Body composition

DEXA (Discovery; Hologic, USA) measured whole-body lean and fat mass (g). A calibration phantom was scanned prior to each test. Participants lay supine with arms pronated. Fat and lean mass indexes were calculated as mass (kg)/height² (m²).

### Statistical analysis

All 384 scans were visually inspected. Outliers (z-score > 3) were reviewed for artefacts; two scans were excluded for soft-tissue analysis due to excess motion. Following outlier removal, normality was assessed using the Shapiro-Wilk test for paired and unpaired data. Potential confounders identified were age, time since surgery, and sex. Limb Symmetry Index (LSI) was calculated as: LSI = (ACLR leg / contralateral uninjured leg) × 100 to aid clinical interpretation. Age and time since surgery were evaluated as potential confounders using two-tailed correlation analyses with each LSI variable, and sex differences were assessed using two-tailed independent-samples *t*-tests. Benjamini-Hochberg procedure was applied to control for false discovery across confounder associations and comparisons.

For the primary hypothesis, one-tailed difference tests were conducted with negative outcomes (e.g. lower MCSA) attributed to the reconstructed leg within the ACLR group. For normally distributed data, paired *t*-tests were conducted. For non-normally distributed data, one-tailed Wilcoxon signed-rank tests were employed. The Holm-Bonferroni adjustment controlled for multiple comparisons at three scan sites: tibia (66%) and femur (20% and 50%), with critical values set at p ≤ 0.017, 0.025, and 0.05 for each variable domain (e.g. MCSA assessed at three sites). For secondary analyses, two-tailed difference tests were conducted comparing LSI between participants with ACLR in the dominant leg versus non-dominant leg. Welch’s *t*-tests were used for normally distributed data, and Mann-Whitney U tests were used for non-normally distributed data. Benjamini-Hochberg procedure was applied to secondary analyses to control for false discoveries.

K-means clustering (k = 2) was used to explore identification of high-integrity recovery (HIR) and low-integrity recovery (LIR) phenotypes. Variables were z-transformed; 0.5% missing data were imputed via multivariate iterative imputation. Principal component analysis (PCA) reduced dimensionality and addressed multicollinearity, explaining 91.2% of the variance. Clustering was performed using the Lloyd algorithm with 10 random starts and 300 iterations. SATA was excluded due to limited contribution to group separation. Hierarchical agglomerative clustering (Ward’s linkage, Euclidean distance) was conducted as an exploratory robustness check. A two-cluster cut was applied to the dendrogram for direct comparison with k-means. Internal clustering validity was evaluated using Silhouette Score, Davies-Bouldin Index (DBI), and Calinski-Harabasz Index (CHI), which evaluate within-cluster cohesion and between-cluster separation to quantify clustering quality. Welch’s one-tailed *t*-tests compared clusters under the directional hypothesis that HIR participants exhibit superior musculoskeletal morphology.

Effect sizes were calculated using methods appropriate to the data distribution and study design. For normally distributed data, Cohen’s *d_z_* was used for matched pairs designs and Hedges’ *g* for independent groups. For non-normally distributed data, the rank-based effect size was used: derived from the Wilcoxon signed-rank test (*r_w_*) for matched pairs and from the Mann-Whitney U test (*r_mw_*) for independent groups. Cohen’s *d_z_* and Hedges’ *g* values of ≥ 0.2, ≥ 0.5, and ≥ 0.8 were interpreted as small, medium, and large effects, respectively. Similarly, *r_w_* and *r_mw_* values of ≥ 0.1, ≥ 0.3, and ≥ 0.5 indicated small, medium, and large effects. 95% CIs were reported for each comparison to describe the magnitude and precision of effects. Statistical analyses and normality testing were conducted using GraphPad Prism (v. 10.4.0; GraphPad Software, USA). Clustering analyses were performed using Python (v. 3.12.7; Python Software Foundation, USA) using the scikit-learn library.

## Results

There were no statistical differences between ACLR and matched controls for age, sex, BMI, FMI, or LMI (Table I). There were no significant associations for age and time since surgery with morphological or mechanical LSI scores and no differences between sexes (Supplementary Material 3).

**Table I.** Participant demographic, anthropometric, and Knee Injury and Osteoarthritis Outcome Scores.

Variable	ACLR	Matched control	Effect size	Two-tailed paired comparison
Biological sex (female:male), n	18:14	18:14	-	-
Median age, yrs (IQR)	25 (21 to 32)	25 (21 to 29)	*r_w_* = 0.24	*W* = 338, p = 0.172
Median BMI, kg/m^2^ (IQR)	24.80 (23.13 to 27.10)	3.50 (22.25 to 27.13)	*r_w_* = 0.26	*W* = 342, p = 0.145
Median fat mass index, kg/m^2^ (IQR)	5.97 (3.92 to 7.26)	5.68 (4.02 to 6.85)	*r_w_* = -0.09	*W* = 237, p = 0.615
Mean lean mass index, kg/m^2^ (SD)	17.86 (2.44)	17.47 (2.54)	*d_z_* = 0.27	*t* = 1.49, p = 0.148
Mean bone mineral density, kg/m^2^ (SD)	1.20 (0.09)	1.18 (0.11)	*d_z_* = 0.12	*t* = 0.70, p = 0.491
Mean time since surgery, mths (SD)	31 (19)	-	-	-
Concomitant meniscus injury, n (%)	18 (56)	-	-	-
Concomitant MCL injury, n (%)	4 (13)	-	-	-
Concomitant LCL injury, n (%)	1 (3)	-	-	-
Hamstring graft, n (%)	25 (78)	-	-	-
Bone-patellar tendon graft, n (%)	7 (22)	-	-	-
Dominant leg injured, n (%)	12 (38)	-	-	-
Median KOOS Pain (0 to 100) (IQR)	94 (90 to 97)	100 (100 to 100)	-	-
Median KOOS Other Symptoms (0 to 100) (IQR)	82 (65 to 93)	96 (93 to 100)	-	-
Median KOOS Activities in Daily Living (0 to 100) (IQR)	100 (97 to 100)	100 (100 to 100)	-	-
Median KOOS Sport and Recreation (0 to 100) (IQR)	85 (75 to 95)	100 (100 to 100)	-	-
Median KOOS Quality of Life (0 to 100) (IQR)	72 (56 to 88)	100 (100 to 100)	-	-

*t* denotes paired *t*-tests; *W* denotes Wilcoxon signed-rank test. Effect sizes are reported as *d_z_* for Cohen’s d for paired *t*-tests, and as rank-based *r_w_* for Wilcoxon signed-rank tests. *p* denotes the probability value used to assess statistical significance.

**Fig. 1:**
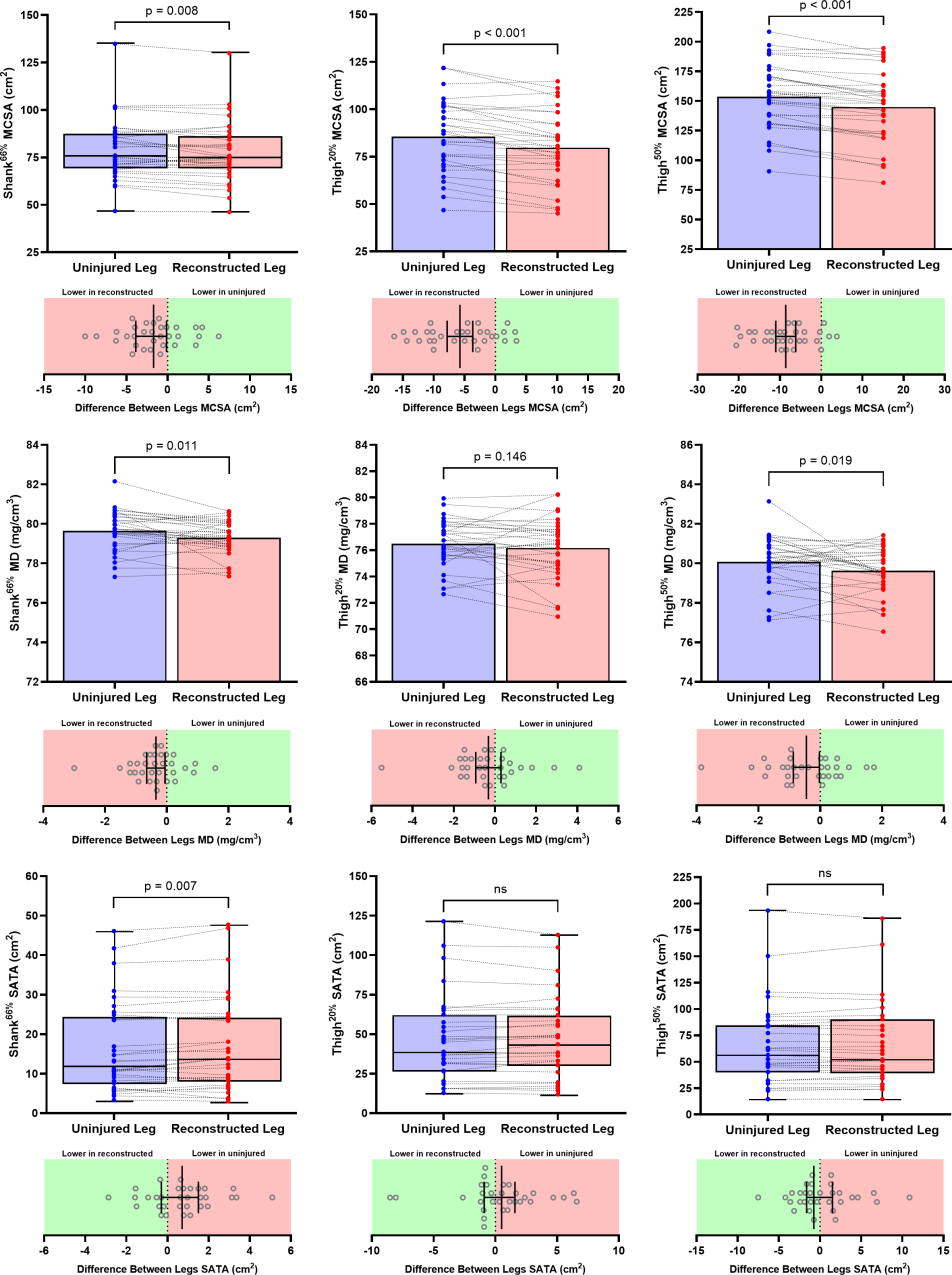
Soft-tissue comparisons between the reconstructed and uninjured contralateral leg. Error bars for difference plots are mean and 95% CI for normally distributed data, and median and 95% CI for non-normally distributed data. MCSA, muscle cross-sectional area; MD, muscle density; SATA, subcutaneous adipose tissue area; ns, not significant.

**Fig. 2:**
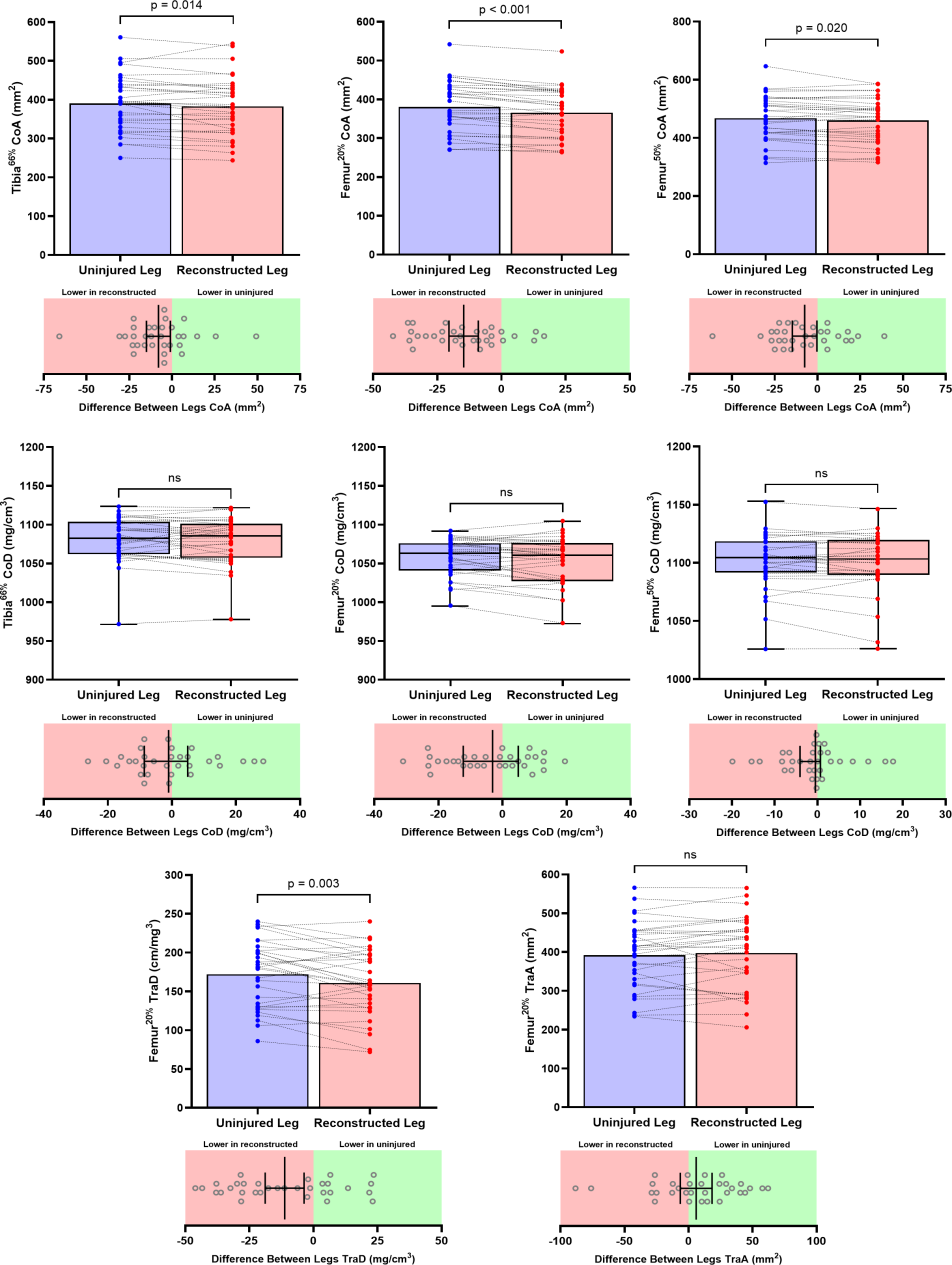
Bone tissue comparisons between the reconstructed and uninjured contralateral leg. Error bars for difference plots are mean and 95% CI for normally distributed data and median and 95% CI for non-normally distributed data. CoA, cortical area; CoD, cortical density; TraA, trabecular area; TraD, trabecular density; ns, not significant.

**Fig. 3:**
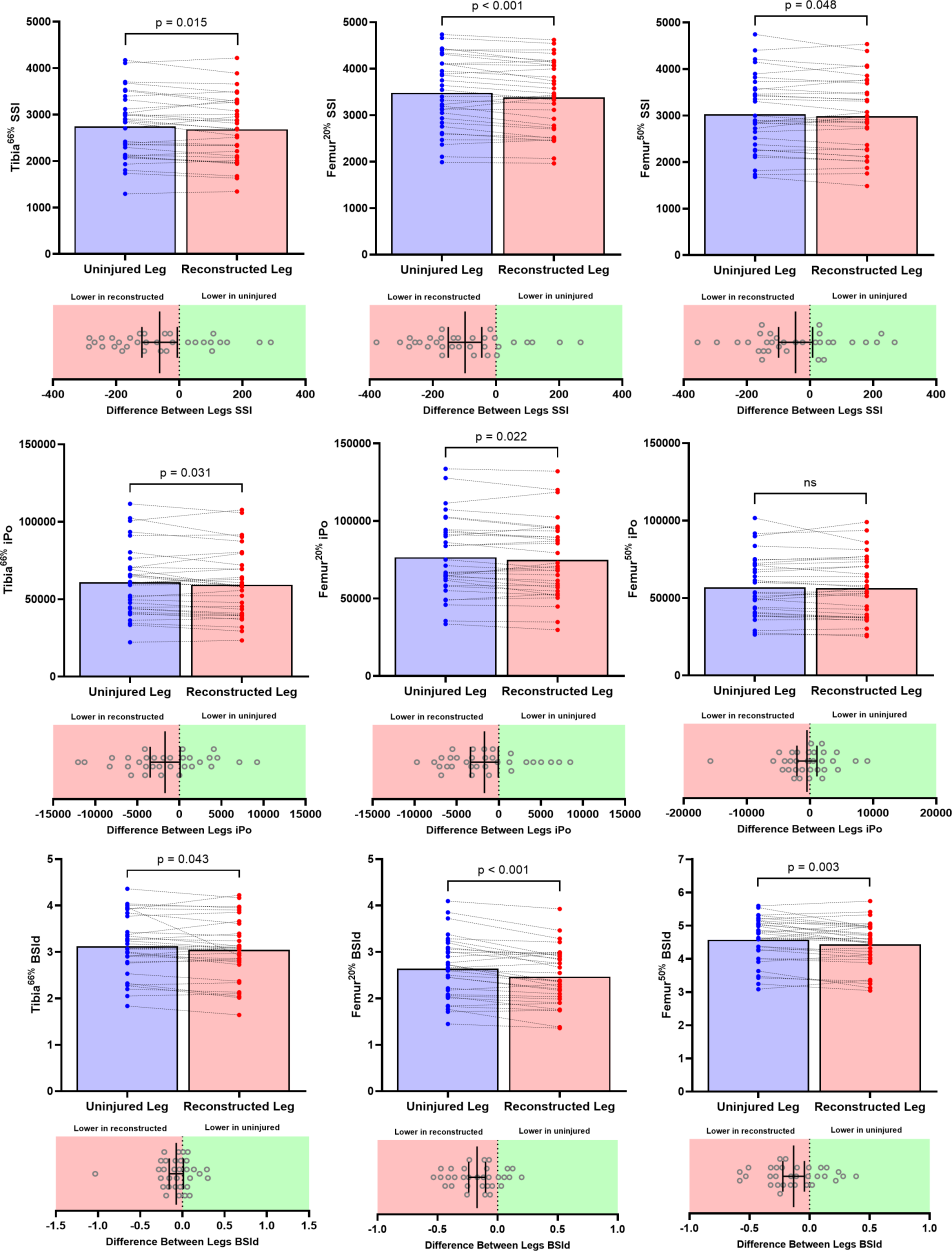
Mechanical properties comparisons between the reconstructed and uninjured contralateral leg. Error bars for difference plots are mean and 95% CI for normally distributed data. BSId, bone strength index for compression; iPo, polar second moment of area; SSI, strength strain index; ns, not significant.

MCSA was significantly lower in the reconstructed leg in comparison to the uninjured leg at shank^66%^, thigh^20%^, and thigh^50%^, with effect sizes ranging from moderate to large (Figure 1, Table II). MD was significantly lower in the reconstructed leg at shank^66%^ and thigh^50%^ with small effects (Figure 1, Table II). SATA was significantly higher in the reconstructed leg at shank^66%^ with a moderate effect, but not at thigh^20%^ or thigh^50%^ (Figure 1, Table II). CoA was significantly lower in the reconstructed leg at all scan sites with small to large effects, however there were no differences in CoD between legs at any scan site (Figure 2, Table II). TraD was significantly lower in the reconstructed leg at femur^20%^, with a moderate effect (Figure 2, Table II). SSI and BSId were significantly lower in the reconstructed leg in comparison to the uninjured control leg at tibia^66%^, femur^20%^, and femur^50%^ (Figure 3, Table II), with small to large effect sizes, but the 95% CI crossed zero for SSI at femur^50%^ and BSId at tibia^66%^. Following multiple comparison adjustment, iPo was not significantly lower at any scan site, despite 95% CI not crossing zero at femur^20%^. LSI scores with 95% CIs which did not cross 100% were observed for MCSA at all scan sites, MD at shank^66%^ and femur^50%^, CoA at all scan sites, TraD at femur^20%^, and SSI, iPo, and BSId at tibia^66%^ and femur^20%^ (Table III).

**Table II.** Anterior cruciate ligament reconstructed group comparisons between the reconstructed and uninjured contralateral leg.

Variable	Scan	Reconstructed	Uninjured contralateral	Test statistics	Effect size
Muscle cross-sectional area, cm^2^	Shank^66%^	74.82 (69.20 to 85.98)	75.65 (69.04 to 87.29)	*W* = 137, 95% CI = -2.94 to -0.31, p = 0.008*	*r_w_* = -0.42
	Thigh^20%^	79.78 (19.14)	85.51 (19.07)	*t*(30) = 5.62, 95% CI = -7.82 to -3.65, p < 0.001*	*d_z_* = -1.01
	Thigh^50%^	144.96 (29.49)	153.57 (28.07)	*t*(30) = 7.27, 95% CI = -11.03 to -6.19, p < 0.001*	*d_z_* = -1.31
Muscle density, mg/cm^3^	Shank^66%^	79.30 (0.87)	79.65 (1.02)	*t*(31) = 2.42, 95% CI = -0.65 to -0.05, p = 0.011*	*d_z_* = -0.43
	Thigh^20%^	76.12 (2.33)	76.48 (1.85)	*t*(30) = 1.07, 95% CI = -0.93 to 0.29, p = 0.146	*d_z_* = -0.19
	Thigh^50%^	79.63 (1.23)	80.07 (1.30)	*t*(30) = 2.16, 95% CI = -0.87 to -0.02, p = 0.019*	*d_z_* = -0.39
Subcutaneous adipose tissue area, cm^2^	Shank^66%^	13.91 (8.32 to 24.44)	12.13 (7.72 to 24.62)	*W* = 394, 95% CI = 0.12 to 1.30, p = 0.007*	*r_w_* = 0.43
	Thigh^20%^	43.65 (30.36 to 62.09)	38.85 (26.91 to 62.42)	*W* = 304, 95% CI = -0.76 to 1.65, p = 0.142	*r_w_* = 0.20
	Thigh^50%^	52.21 (39.43 to 90.33)	56.43 (40.37 to 84.91)	*W* = 236, 95% CI = -1.09 to 1.60, p = 0.478	*r_w_* = -0.04
Cortical area, mm^2^	Tibia^66%^	382.91 (75.91)	390.84 (72.70)	*t*(31) = 2.31, 95% CI = -14.92 to -0.93, p = 0.014*	*d_z_* = -0.41
	Femur^20%^	365.90 (63.43)	380.62 (67.61)	*t*(31) = 5.26, 95% CI = -20.42 to -9.01, p < 0.001*	*d_z_* = -0.93
	Femur^50%^	460.12 (78.13)	467.65 (79.19)	*t*(31) = 2.14, 95% CI = -14.71 to -0.34, p = 0.020*	*d_z_* = -0.38
Cortical density, mg/cm^3^	Tibia^66%^	1,085.35 (1057.41 to 1,101.08)	1,082.60 (1,062.08 to 1,103.76)	*W* = 221, 95% CI = -5.63 to 3.70, p = 0.216	*r_w_* = 0.14
	Femur^20%^	1,061.33 (1,027.79 to 1,076.91)	1,063.75 (1,041.46 to 1,076.52)	*W* = 178, 95% CI = -9.18 to 0.40, p = 0.055	*r_w_* = -0.28
	Femur^50%^	1,103.04 (1,089.48 to 1,119.41)	1,104.33 (1,091.38 to 1,118.24)	*W* = 209, 95% CI = -3.92 to 1.78, p = 0.156	*r_w_* = -0.18
Trabecular area, mm^2^	Femur^20%^	397.66 (92.32)	391.67 (86.82)	*t*(31) = 0.98, 95% CI = -6.42 to 18.40, p = 0.166	*d_z_* = 0.17
Trabecular density, mg/cm^3^	Femur^20%^	160.93 (42.50)	172.15 (42.76)	*t*(31) = 3.02, 95% CI = -18.81 to -3.64, p = 0.003*	*d_z_* = -0.53
Strength strain index	Tibia^66%^	2,683.93 (699.91)	2,746.37 (710.42)	*t*(31) = 2.27, 95% CI = -118.54 to -6.34, p = 0.015*	*d_z_* = -0.40
	Femur^20%^	3,382.79 (757.10)	3,480.62 (774.78)	*t*(31) = 3.76, 95% CI = -150.88 to -44.78, p < 0.001*	*d_z_* = -0.66
	Femur^50%^	2,983.66 (803.08)	3,029.00 (805.13)	*t*(31) = 1.72, 95% CI = -99.19 to 8.51, p = 0.048	*d_z_* = -0.30
Polar second moment of area	Tibia^66%^	59,308.64 (21,709.55)	61,003.25 (21,891.06)	*t*(31) = 1.94, 95% CI = -3472.59 to 83.39, p = 0.031	*d_z_* = -0.34
	Femur^20%^	74,883.05 (24,650.32)	76,587.33 (24,864.54)	*t*(31) = 2.10, 95% CI = -3360.76 to -47.78, p = 0.022	*d_z_* = -0.37
	Femur^50%^	56,411.94 (19,872.87)	56,870.45 (19,516.02)	*t*(31) = 0.59, 95% CI = -2040.00 to 1122.97, p = 0.279	*d_z_* = -0.10
Bone strength index for compression	Tibia^66%^	3.05 (0.67)	3.12 (0.64)	*t*(31) = 1.78, 95% CI = -0.16 to 0.01, p = 0.043	*d_z_* = -0.31
	Femur^20%^	2.47 (0.61)	2.64 (0.66)	*t*(31) = 4.95, 95% CI = -0.24 to -0.10, p < 0.001*	*d_z_* = -0.88
	Femur^50%^	4.44 (0.68)	4.57 (0.68)	*t*(31) = 3.01, 95% CI = -0.22 to -0.04, p = 0.003*	*d_z_* = -0.53

Data are presented as mean (SD), median (IQR). *t* denotes paired *t*-tests;* W* denotes Wilcoxon signed-rank test. Effect sizes are reported as* d_z_* for Cohen’s d for paired *t*-tests, and as rank-based* r_w_* for Wilcoxon signed-rank tests. *p* denotes the probability value used to assess statistical significance. The Holm-Bonferroni adjustment controlled for multiple comparisons at three scan sites: tibia (66%) and femur (20% and 50%), with critical values set at p ≤ 0.017, 0.025, and 0.05 for each variable domain (e.g., muscle cross-sectional area assessed at three sites). Multiple comparison adjustments were not calculated for trabecular variables as these were measured at a single site (no multiple comparisons within domain). All test statistics were based on one-tailed analyses.

* Statistically significant result after adjustment and 95% CI not crossing zero.

MCSA, muscle cross-sectional area; SATA, subcutaneous adipose tissue area.

**Table III.** Anterior cruciate ligament reconstructed group comparisons between the reconstructed and uninjured contralateral leg.

Variable	Scan location	Mean limb symmetry index score, % (SD)
Muscle cross-sectional area, cm^2^	Shank^66%^	97.95 (4.62), 95% CI = 96.35 to 99.55*
	Thigh^20%^	93.11 (6.62), 95% CI = 90.78 to 95.44*
	Thigh^50%^	94.06 (4.71), 95% CI = 92.41 to 95.72*
Muscle density, mg/cm^3^	Shank^66%^	99.57 (1.02), 95% CI = 99.21 to 99.92*
	Thigh^20%^	99.59 (2.19), 95% CI = 98.82 to 100.36
	Thigh^50%^	99.45 (1.42), 95% CI = 98.95 to 99.95*
Subcutaneous adipose tissue area, cm^2^	Shank^66%^	104.54 (16.32), 95% CI = 98.88 to 110.19
	Thigh^20%^	101.93 (7.04), 95% CI = 99.45 to 104.41
	Thigh^50%^	99.44 (4.99), 95% CI = 97.69 to 101.20
Cortical area, mm^2^	Tibia^66%^	97.87 (4.70), 95% CI = 96.24 to 99.49*
	Femur^20%^	96.30 (4.15), 95% CI = 94.86 to 97.74*
	Femur^50%^	98.45 (4.15), 95% CI = 97.01 to 99.89*
Cortical density, mg/cm^3^	Tibia^66%^	99.92 (1.20), 95% CI = 99.50 to 100.33
	Femur^20%^	99.58 (1.27), 95% CI = 99.14 to 100.02
	Femur^50%^	99.90 (0.73), 95% CI = 99.65 to 100.15
Trabecular area, mm^2^	Femur^20%^	102.76 (9.68), 95% CI = 99.40 to 106.11
Trabecular density, mg/cm^3^	Femur^20%^	93.73 (12.88), 95% CI = 89.27 to 98.19*
Strength strain index	Tibia^66%^	97.83 (5.47), 95% CI = 95.94 to 99.73*
	Femur^20%^	97.27 (4.35), 95% CI = 95.76 to 98.78*
	Femur^50%^	98.43 (4.96), 95% CI = 96.71 to 100.15
Polar second moment of area	Tibia^66%^	97.24 (7.31), 95% CI = 94.71 to 99.78*
	Femur^20%^	97.74 (6.31), 95% CI = 95.56 to 99.93*
	Femur^50%^	98.97 (6.57), 95% CI = 96.70 to 101.25
Bone strength index for compression	Tibia^66%^	97.60 (6.81), 95% CI = 95.24 to 99.96*
	Femur^20%^	93.81 (7.08), 95% CI = 91.36 to 96.27*
	Femur^50%^	99.09 (6.22), 95% CI = 96.93 to 101.24

* 95% CI not crossing 100%.

There were significantly lower LSI for MD thigh^50%^ and TraD femur^20%^ between the ACLR dominant leg and the ACLR non-dominant leg groups, and large effects were detected (Supplementary Material 4). In the uninjured group, MCSA was significantly lower at thigh^20%^ and thigh^50%^ in the non-dominant leg in comparison to the dominant leg, with small effects detected (Supplementary Material 5). There were no statistical differences in MD, SATA, CoA, CoD, TraA, TraD, SSI, iPo, and BSId at any scan site between the dominant and non-dominant leg (Supplementary Material 5).

**Fig. 4:**
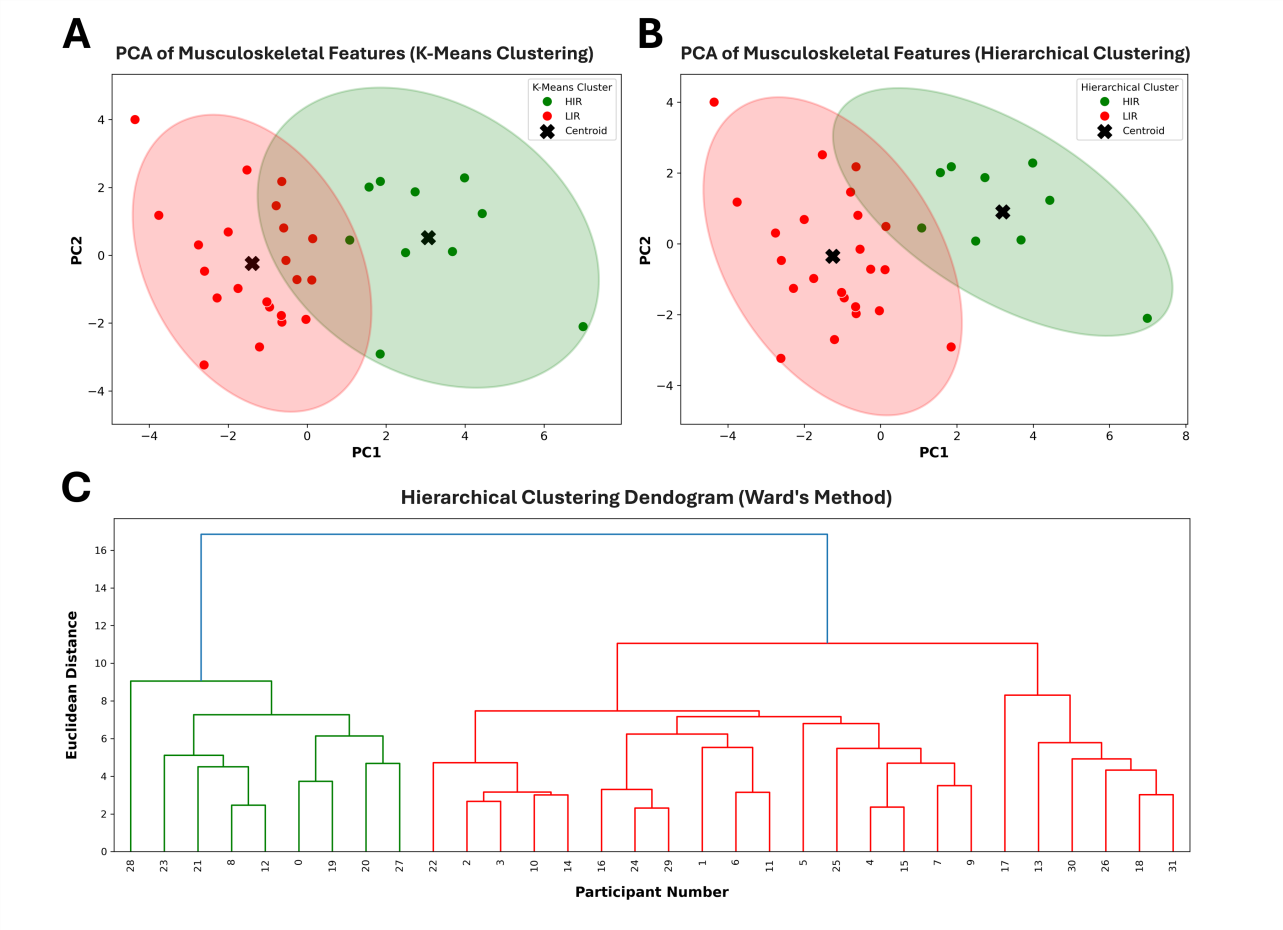
a) K-means clustering (k = 2) visualized in principal component analysis (PCA) space, showing separation into high-integrity recovery (HIR, red) and low-integrity recovery (LIR, green) phenotypes. Ellipses represent 95% CIs, and centroids are marked with black crosses. b) Hierarchical clustering (Ward’s method, k = 2) visualized in PCA space, demonstrating a similar separation into HIR and LIR clusters. c) Dendrogram of hierarchical clustering (Ward’s method), illustrating the two-cluster solution (blue linkage) based on Euclidean distance.

Exploratory K-means clustering of LSI scores of muscle, bone, and mechanical property variables identified two distinct phenotypes: a high integrity recovery and a low integrity recovery cluster (Figure 4). The K-means two-cluster solution yielded a silhouette score of 0.23, a DBI of 1.59 indicating acceptable separation, and a CHI of 9.81 reflecting moderate between-cluster dispersion. Hierarchical clustering produced a comparable solution, with a silhouette score of 0.23, DBI of 1.55, and CHI of 9.41. K-means clustering assigned ten participants (31%) to the HIR cluster and 22 (69%) to the LIR cluster. Hierarchical clustering similarly classified nine participants (28%) as HIR and 23 (72%) as LIR. Overall, 94% of participants were classified into the same cluster by both methods. Cluster visualizations in PCA space demonstrated clear qualitative separation (Figure 4). Soft-tissue, bone, and mechanical integrity were statistically significantly lower in the K-Means LIR than the HIR cluster, with no differences in leg dominance, sex, or time since injury (Supplementary Material 6).

## Discussion

This study’s key finding is the reduced capacity of the ACLR leg to resist compressive and torsional forces at tibia^66%^, femur^20%^, and femur^50%^ compared to the uninjured contralateral leg. Femur^20%^ demonstrated the largest deficit for resistance to compressive forces. Across scan sites, the deficits were more pronounced for compression than rotation. These mechanical deficits appear to be driven, at least in part, by alterations in bone geometry and density. CoA was consistently lower at all sites, and TraD was reduced at femur^20%^ in the reconstructed leg, indicative of a compromised bone structure. This potentially creates a scenario where continued exposure to joint loading in a structurally compromised femur and tibia reduces the capacity to transmit and dissipate mechanical loads, increasing stress on adjacent structures such as cartilage, menisci, and subchondral bone, which theoretically could contribute to accelerating degenerative changes and elevated risk of PTOA. Crucially, these deficits persist during daily loading walking and may be further exacerbated during return to high-impact sporting activities. Of note, no asymmetries were observed between dominant and non-dominant legs in the control group, reinforcing the idea that these differences are not due to natural limb dominance, but rather to ACLR-related maladaptations.

The spatial pattern of bone loss in our study, observed most notably at femur^20%^, distal to the articular surface, highlights the complexity of skeletal responses to altered loading. In a population predominately experiencing knee OA, sclerosis of the subchondral bone in regions immediately adjacent to cartilage was positively associated with joint degeneration.^20^ However, our findings suggest that in regions further from the joint line, bone resorption may dominate. This may reflect an earlier, disuse-driven adaptation that occurs in more metaphyseal bone regions and precedes or occurs concomitantly with cartilage loss. The aetiology of our results may be related to mechanical underloading, which can persist in the long term after early postoperative phase. Of concern is underloading of the reconstructed limb during walking, which has been associated with joint tissue degeneration.^35^ Cortical thinning, likely due to endosteal resorption in response to disuse, may explain the lower cortical area, while changes in cortical density are typically slower to manifest.^36^ Trabecular bone, being more metabolically active, is more rapidly remodelled and is consequently more sensitive to changes in loading.^36^ While asymmetry at femur^50%^ is more distal from the knee joint than the femur^20%^ scan site, given that mechanical forces are transferred through the femur during high-impact activities, even mid-shaft cortical deficits may have implications for PTOA development. Another potential consequence of bone morphology alterations is that reductions in muscle size and quality may diminish the mechanical forces placed on bone, impairing mechanotransductive signalling critical for bone maintenance.

MCSA was consistently lower in the reconstructed leg compared with the contralateral side. Prior studies report reduced muscle size and volume in the reconstructed thigh.^23^ Quadriceps MCSA remained reduced up to 289 weeks, while hamstring volume deficits appeared stable from 26 to 212 weeks.^23^ Although previous work has typically focused on the thigh, recent evidence also shows lower gastrocnemius volume in the ACLR leg,^37^ which aligns with our findings. While deficit magnitude was smaller than in the thigh, our results suggest that long-term impairments may extend below the knee.

Our results also suggest lower MD in both the shank and mid-thigh, indicating greater intramuscular fat infiltration. This partially aligns with prior studies, but contrasts with others. One longitudinal study found no differences in quadriceps quality between pre-ACLR and one-year post-surgery, but did observe increased intramuscular fat in the hamstrings.^38^ In contrast, a separate study conducted five years post-ACLR observed no differences in quadriceps echo intensity between reconstructed, contralateral, and control legs.^39^ We also observed significantly higher SATA in the reconstructed leg at shank^66%^ only, which contrasts with earlier separate evidence demonstrating 9% higher subcutaneous fat at the thigh in the reconstructed leg compared with the contralateral leg 13 months post-ACLR.^40^ These mixed findings may reflect differences in the sensitivity of imaging methods to soft-tissue adaptations, and underscore the need for longitudinal studies using MRI to clarify post-ACLR muscle quality changes, particularly across individual musculature.

Another novel finding was that the dominant leg in the uninjured control group had significantly greater MCSA in the thigh at both scan sites. Leg dominance may influence the extent of muscle atrophy following ACLR, particularly if the injured leg was dominant, potentially masking true asymmetries. This is supported by significantly higher MCSA at thigh^20%^ and thigh^50%^ in the dominant leg of control participants. Of note is the observation of similar patterns in the ACLR population, where MD at thigh^50%^ and TraD at femur^20%^ were significantly higher in those who injured their dominant leg. However, this interpretation requires caution, given the small sample sizes for group-wise comparisons.

Ultimately, this soft-tissue maladaptation observed on average across the ACLR population may contribute to knee extensor weakness. Reduced muscle function is theorized to result in aberrant knee biomechanics, where compromised function may be unable to adequately attenuate knee joint loading, resulting in excessive stress on joint tissues.^41^ Supporting this, two studies report moderate to strong associations between muscle size and knee extensor strength in individuals between six months to several years post-ACLR.^42^ In another study, whole quadriceps cross-sectional area was significantly related to peak isometric knee extension torque, whereas activation deficits were not, at seven months post-ACLR.^10^ Furthermore, a lower muscle density in the ACLR leg may increase the risk of PTOA development. A longitudinal study in 69 middle-aged people with and without OA demonstrated that greater intramuscular quadriceps and vastus medialis fat was associated with higher knee degeneration scores over three years, after adjusting for age, sex, and BMI.^16^ Theoretically, reduced muscle density driven through intramuscular fat infiltration may contribute to PTOA pathogenesis by reducing the muscles’ capacity to absorb impact loads, considering the association between force production and muscle quality,^14^ and perhaps through local oxidative stress or low-grade inflammation via adipokine and pro-inflammatory cytokine release, which could further impair bone and joint tissue metabolism. Given the potential associations between morphology and PTOA risk, understanding how ACL injury impacts both bone and soft-tissue is needed to inform future interventions.

The causal mechanisms of soft-tissue maladaptation following ACLR are yet to be definitively elucidated. However, long periods of non-weightbearing and then sustained periods of partial weightbearing may induce disuse atrophy and reduced muscle quality. In uninjured populations, muscle size declines and intramuscular fat increases have been observed after just zero to seven days to four weeks of bed rest or limb suspension.^43,44^ A contributing molecular factor may be myostatin, a negative regulator of muscle growth, which shows increased mRNA expression in quadriceps following ACLR and during short-term disuse.^45,46^ Elevated concentrations have been observed pre- and postoperatively in other surgical contexts,^47^ supporting its potential role in disuse-induced atrophy. Complementing this are findings from ex vitro studies indicating greater expression of myostatin mRNA,^45^ and lower quantity of satellite cells in quadricep muscle biopsy samples in comparison to the contralateral leg shortly after injury.^48^

Further supporting the role of disuse, vastus lateralis biopsies collected both shortly after ACL injury and in the years following reconstruction show preferential atrophy of type IIa/x fibres, indicating disuse as a primary mechanism of muscle loss.^40,48,49^ Similarly, selective atrophy of type II fibres has been observed in 68% of patients with end-stage osteoarthritis, also attributed to disuse.^50^ Regular physical activity is positively associated with muscle quality,^51,52^ and higher physical activity levels correspond with more favourable muscle density ratios between limbs at 12 months post-ACLR.^38^ Interestingly, this association appears independent of total body fat, as BMI was relatively consistent across participants.^38^ In line with this, individuals assessed approximately four years after ACLR showed no difference in muscle quality between the reconstructed limb and equally active controls, suggesting that long-term physical activity engagement may help to preserve muscle quality post-reconstruction.^39^

Clustering analysis revealed two distinct recovery phenotypes. The majority of participants (69%) were assigned to the LIR cluster, characterized by significantly lower MCSA, MD, CoA, TraA, TraD, and mechanical property LSI in the reconstructed leg. This musculoskeletal profile reflects compromised muscle mass and quality, cortical and trabecular bone structure, and impaired load-bearing capacity. Identifying this at-risk phenotype may enable early intervention strategies aimed at restoring musculoskeletal integrity and protecting joint health. Future longitudinal research is needed to confirm whether individuals within this low-integrity recovery phenotype indeed experience greater structural decline or clinical symptoms associated with PTOA over time. However, if a multitissue clustering approach can provide insights into those at risk of PTOA, a combination of targeted rehabilitation, medical devices, and pharmacological approaches may help to mitigate the risk of PTOA development.^53^

While the strengths of this study include a comprehensive quantification of limb morphology, and a participant population consisting predominantly of females, who are at higher risk of sustaining an ACL injury,^54^ there are several limitations. First, a cross-sectional study design was used, which limits the strength of inferences we can make on the effect of ACL injury and reconstruction on the affected leg morphology. Future longitudinal studies are warranted to confirm whether the LIR cluster predicts progressive PTOA features, such as increased cartilage T2 relaxation time, meniscal degeneration, or worsening pain scores. Furthermore, the study population consists of a heterogenous time since injury incidence and ligament reconstruction. In acknowledging this limitation, statistical assessment of confounders was conducted, and there appeared to be limited impact on the data. Considering the small sample size, we did not test for difference across graft types. We acknowledge the influence of different graft types on potential soft-tissue deficits, given that knee extensor weakness following bone-patella tendon grafts and knee flexor weakness following hamstring grafts have been reported.^11^ Future studies with a larger population are required to conduct stratified analyses by graft type to explore this potential confounder. Further, 56% sustained concomitant meniscus damage, which may influence our results considering worse outcomes observed following ACL injury.^55^ Another limitation is the lack of sensitivity in delineating individual muscles. Future research should seek to measure individual muscles longitudinally. Furthermore, while bone strength indices derived from pQCT are validated surrogates,^34^ they are not direct measures of failure load or material properties. Additionally, detailed participant rehabilitation histories were not available; however, all underwent supervised rehabilitation consistent with standard clinical practice in the UK, which could be considered a strength considering the potential heterogeneity in post-injury and post-surgery rehabilitation regimen. A final consideration is that the study population did not include elite or professional athletes; however, this arguably improves its external validity, considering the generalizability to active adults rather than elite athletes only.

In conclusion, this is the first study to comprehensively assess soft-tissue, bone tissue, and mechanical properties at multiple scan sites in the calf and thigh, one to six years following ACLR. On average, individuals post-ACLR exhibit persistent muscle atrophy, intra-muscular fat infiltration, reduced cortical area and trabecular density, and impaired mechanical properties in the reconstructed leg compared with the contralateral limb, which theoretically may increase PTOA risk. Finally, we successfully clustered individuals into high- and low-integrity recovery phenotypes, offering a framework for stratifying PTOA risk.

## Data Availability

The data that support the findings for this study are available to other researchers from the corresponding author upon reasonable request.
